# Role of serum periostin in severe obstructive sleep apnea with albuminuria: an observational study

**DOI:** 10.1186/s12931-020-01413-0

**Published:** 2020-06-09

**Authors:** Hironobu Sunadome, Hisako Matsumoto, Ryo Tachikawa, Takeshi Matsumoto, Kiminobu Tanizawa, Toru Oga, Junya Ono, Shoichiro Ohta, Kenji Izuhara, Toyohiro Hirai, Kazuo Chin

**Affiliations:** 1 Department of Respiratory Medicine, Graduate School of Medicine, Kyoto University, 54 Kawaharacho, Shogoin, Sakyo-ku, Kyoto city, Kyoto prefecture 606-8507 Japan; 2 Department of Respiratory Care and Sleep Control Medicine, Graduate School of Medicine, Kyoto University, 54 Kawaharacho, Shogoin, Sakyo-ku, Kyoto City, Kyoto prefecture 606-8507 Japan; 3 Shino-Test Corporation, 2-29-4 Oonodai, Minami-ku, Sagamihara City, Kanagawa prefecture 252-0331 Japan; 4 Department of Laboratory Medicine, Saga Medical School, 5-1-1 Nabeshima, Saga City, Saga prefecture 840-8502 Japan; 5 Division of Biochemistry, Department of Biomolecular Science, Saga Medical School, 5-1-1 Nabeshima, Saga City, Saga prefecture 840-8502 Japan

**Keywords:** Obstructive sleep apnea, Body mass index, Serum periostin, Albuminuria

## Abstract

**Background:**

Periostin is a matricellular protein and is a useful marker in respiratory diseases. However, the roles of periostin in patients with obstructive sleep apnea (OSA) remain unclear. Several in vitro studies have suggested that mechanical stress, hypoxia, impaired metabolism, and kidney injury, which often accompany OSA, may upregulate the expression of periostin. Meanwhile, serum periostin level has been negatively associated with body mass index (BMI) in the general population. In this study, we hypothesized that a high level of serum periostin despite being overweight/obese may discriminate severe OSA or OSA with comorbidities from mild OSA with obesity alone. We aimed to clarify the roles of periostin in patients with OSA to assist in elucidating the heterogeneity of OSA with comorbidities.

**Methods:**

Among patients diagnosed as OSA, we examined the associations between serum periostin levels and clinical indices, including the severity of OSA, BMI, and comorbidities, using a multifaceted approach. The serum periostin levels and clinical indices were assessed after 3 months of continuous positive airway pressure (CPAP) treatment.

**Results:**

In 96 patients with OSA, serum periostin level was negatively correlated with BMI, albeit marginally, and tended to be higher in severe OSA than in others when adjusted for BMI. Cluster analysis identified four clusters, including two severe OSA clusters, one of which was characterized by high serum periostin levels and the presence of comorbidities, including albuminuria. In a comparative analysis of severe OSA cases (*n* = 53), the level of serum-free fatty acids and the frequency of albuminuria were higher in patients with high serum periostin level of ≥87 ng/mL, which was the highest quintile among all participants, than in those with low serum periostin levels (< 87 ng/mL, *n* = 41). In patients with severe OSA and high serum periostin levels, the levels of serum periostin and urinary albumin significantly decreased after 3 months of CPAP treatment.

**Conclusions:**

Elevated serum periostin in patients with OSA despite being overweight/obese may be an indicator of severe OSA with comorbidities, particularly albuminuria.

## Background

Obstructive sleep apnea (OSA) is a major public health concern and has been to shown to increase in prevalence with obesity, which is its most important risk factor [1]. OSA often coexists with several respiratory and nonrespiratory diseases, including chronic obstructive pulmonary disease (COPD), asthma [2–4], abnormal glycolipid metabolism, cardiovascular diseases [1], fatty liver [5], and kidney injury [6]. OSA has deleterious effects on these comorbidities by causing mechanical stress on airways, oxidative stress by intermittent hypoxia (IH), and interrupted sleep, all of which eventually increase the risks for poor outcomes. Besides comorbidities, OSA has recently been considered a heterogeneous disease that comprises several phenotypes with different symptoms, comorbidities, and responsiveness to continuous positive airway pressure (CPAP) treatment [7–9]. Therefore, to improve the management of OSA, recognition of comorbidities and heterogeneity in OSA is required.

Serum periostin, which is an extracellular matrix protein, is a robust marker of T helper type 2 (Th2) inflammation, particularly in asthma [10–12]. Interestingly, several recent studies on asthma and the general population have revealed that serum periostin was negatively associated with body mass index (BMI) [12–16] and serum leptin [15]. In addition to Th2 inflammation, periostin has been shown by in vitro studies to be upregulated under several stimulations, including mechanical stress [17] and hypoxic stimulation [18], which are major features of OSA [19]. Furthermore, periostin was suggested to play crucial roles in epithelial mesenchymal transition and lung fibrosis, both of which have been correlated with hypoxia pathways [20, 21]. In addition, as obesity develops, the macrophages in visceral adipose tissue were demonstrated to secrete periostin, possibly in response to hypoxia [22]. Other studies reported that the expression of periostin was upregulated in fatty liver tissues [23] and in the renal tissues of a kidney injury mouse model [24].

These findings suggested that the serum periostin levels in patients with OSA may reflect a combination of its downregulation by obesity and upregulation by hypoxic stimulation or metabolic comorbidities. Therefore, we hypothesized that high serum periostin levels in patients with OSA, despite being overweight/obese, may discriminate severe OSA or OSA with comorbidities from mild OSA with obesity alone. To test this hypothesis, we aimed to clarify the associations between serum periostin levels and the sleep metrics of OSA and its comorbidities, including dyslipidemia or kidney injury, which have not been examined previously. This may assist in understanding the heterogeneity of OSA with comorbidities.

## Methods

### Study participants

The participants were recruited from a cohort of clinically stable patients aged between 20 and 80 years and who were diagnosed as OSA based on the findings of overnight polysomnography (PSG) at the Sleep Laboratory of Kyoto University Hospital between March 2014 and April 2015. Patients with dominant central sleep apnea, history of previous treatment for OSA, premenopausal status, hemodialysis or uncontrolled comorbidity, or ongoing systemic corticosteroid treatment were excluded, as described previously [25].

This study was approved by the ethics committee of Kyoto University and was registered in the UMIN Clinical Trials Registry (Registry ID UMIN000012639). Written informed consent was obtained from all participants.

#### Polysomnography

Overnight PSG was performed between 22:00 and 06:00 using standard techniques, as described previously [25]. Apnea was defined as an airflow reduction of > 90% for > 10 s, and hypopnea was identified as an airflow reduction of > 30% for > 10 s accompanied by > 3% oxygen desaturation or arousal [26]. The definitions of severity of OSA, high cumulative percentage of sleep time with percutaneous oxygen saturation < 90% (CT_90_), high (≥3%) oxygen desaturation index (3% ODI), and high arousal index (AI) are described in the Supplementary Methods (Additional file 1). Participants with apnea–hypopnea index (AHI) of ≥20 were treated with CPAP according to the health insurance system in Japan.

#### Assessments

Assessments were performed on the first diagnostic PSG (baseline) and on the follow-up PSG 3 months after CPAP initiation. Patient history was taken at baseline. Physical examinations, blood tests, and urinalysis were performed at baseline and on follow-up PSG. Blood samples were collected between 06:30 and 07:00 after the completion of PSG. Sample analyses included blood glucose and serum levels of insulin, lipids, and periostin. Serum periostin levels were measured using an enzyme-linked immunosorbent assay at Shino-Test Corporation (Kanagawa, Japan). Using a 12-h overnight urine sample, urinalysis and analysis of urine albumin and creatinine were performed [27]. The degree of albuminuria was determined by the urine albumin (mg)–creatinine (g) ratio (UACR), as follows: microalbuminuria for UACR of > 20 mg/g in men and > 30 mg/g in women and macroalbuminuria for UACR of > 300 mg/g [28]. The definitions of other comorbidities, including hypertension, dyslipidemia, diabetes mellitus (DM), COPD, asthma, and allergic rhinitis, are described in the Supplementary Methods (Additional file 1).

The associations between serum periostin levels and the severity of OSA; sleep metrics, including CT_90_, 3% ODI, and AI; and the other clinical indices were evaluated. Changes in the measurements from baseline to after 3 months of CPAP therapy were examined.

### Statistical analysis

Statistical analyses were performed using JMP® version 12 (SAS Institute, Tokyo, Japan). Comparisons between two or more groups were performed using t-test, χ^2^ test, or Wilcoxon rank sum test, as appropriate. To determine correlations between continuous variables, the linear least squares method was used. For statistical analysis, the common logarithm of the serum periostin level was used. To assess the associations among serum periostin, severe OSA, and comorbidities, cluster analysis was performed. The detailed methods are described in the Supplementary Methods (Additional file 1). The interactions of serum periostin levels with sleep metrics and clinical indices were assessed. Finally, changes in the paired variables after CPAP treatment were examined using paired t-test, Wilcoxon signed-rank test, or McNemar test, as appropriate. Data are presented as the mean ± SD, and *p* ≤ 0.05 was considered to indicate statistical significance.

## Results

### Participant characteristics

**Fig. 1:**
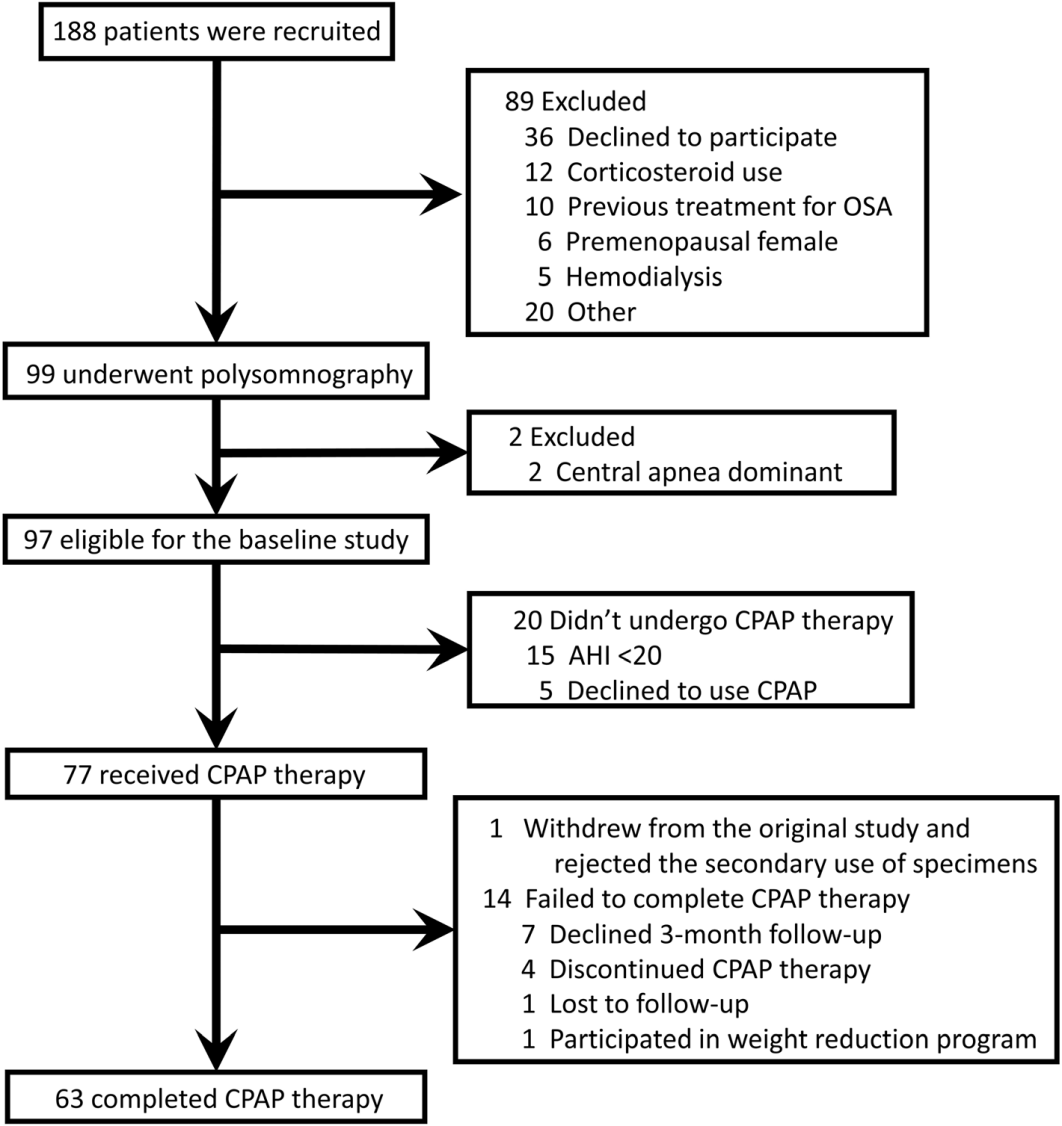
Flowchart of the study

Of 188 patients in total, 97 met the inclusion criteria but 1 withdrew from the original study and rejected the secondary use of specimens [25]. Therefore, the final analyses were conducted for 96 patients (Fig. 1). The characteristics of the patients are presented in Table 1. The mean serum periostin level was 67.3 ± 22.8 ng/mL, which was equivalent to the average of the general population in Japan [15]. Among 96 subjects, 86 had at least one of the major comorbidities, such as DM, hypertension, dyslipidemia, albuminuria, COPD, asthma, or allergic rhinitis.

**Table 1:** Patient characteristics

**Index**	
**Clinical background**	
Age, years	56 ± 11
Sex, male/female, n	74/22
BMI, kg/m^2^	27.6 ± 4.2
Abdominal circumference, cm	94.8 ± 20.7
Smoking, current/others, n	21/75
**Sleep parameters**	
AHI, events/h	36.5 ± 20.7
CT_90_, %	13.1 ± 18
Lowest oxygen saturation, %	78.9 ± 8.3
3% ODI, events/h	35.3 ± 20.5
Arousal index, events/h	34.4 ± 17.8
OSA, mild/moderate/severe^a^, n	12/31/53
**Comorbidities**	
Diabetes mellitus, +/−, n	26/70
Insulin use, +/−, n	5/91
Hypertension, +/−, n	58/38
Hypertension (self-reported), +/−, n	65/31
Dyslipidemia, +/−, n	62/34
Albuminuria^b^, macro/micro/−, n	2/12/82
Chronic obstructive pulmonary disease, +/−, n	2/94
Asthma, +/−, n	4/92
Allergic rhinitis, +/−, n	16/80
**Blood/Urine tests**	
Total cholesterol, mg/dL	196 ± 34
High-density lipoprotein, mg/dL	53 ± 15
Low-density lipoprotein, mg/dL	117 ± 30
Triglycerides, mg/dL	172 ± 121
Free fatty acids, mg/dL	490 ± 160
Blood glucose, mg/dL	101 ± 24
Hemoglobin A1c, %	6.2 ± 0.9
Insulin, μU/mL	10.7 ± 10.2
Serum periostin, ng/mL	67.3 ± 22.8
Urinary albumin–creatinine ratio^c^, mg/g	25.4 ± 107.1

Baseline data are presented as mean ± SD or number

^a^Severity of OSA was defined on the basis of the AHI, as follows: mild, ≥5 to < 15;

moderate, ≥15 to < 30; or severe, AHI ≥30

^b^Defined as positive if the urinary albumin–creatinine ratio was ≥20 mg/g in men or ≥ 30 mg/g in women. ^c^Defined as urine albumin/creatinine

*BMI* body mass index, *AHI* apnea–hypopnea index, *CT*_*90*_ cumulative percentage of sleep time with percutaneous oxygen saturation < 90%, *ODI* oxygen desaturation index

### Associations between serum periostin level and clinical variables in OSA: BMI, disease severity, sleep metrics, and other clinical indices

**Fig. 2:**
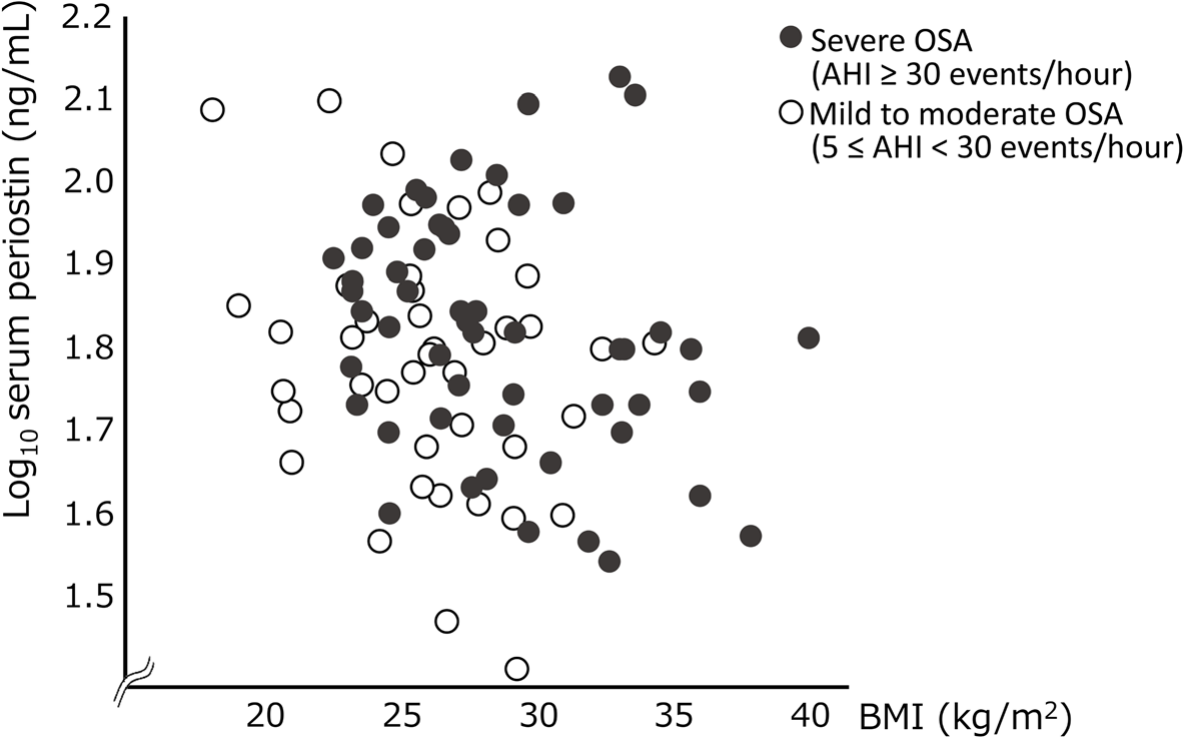
Associations between BMI and serum periostin at baseline. Black circles represent patients with severe OSA (AHI ≥30), and white circles represent patients with mild to moderate OSA (AHI ≥5 to < 30). BMI, body mass index; OSA, obstructive sleep apnea; AHI, apnea–hypopnea index

**Fig. 3:**
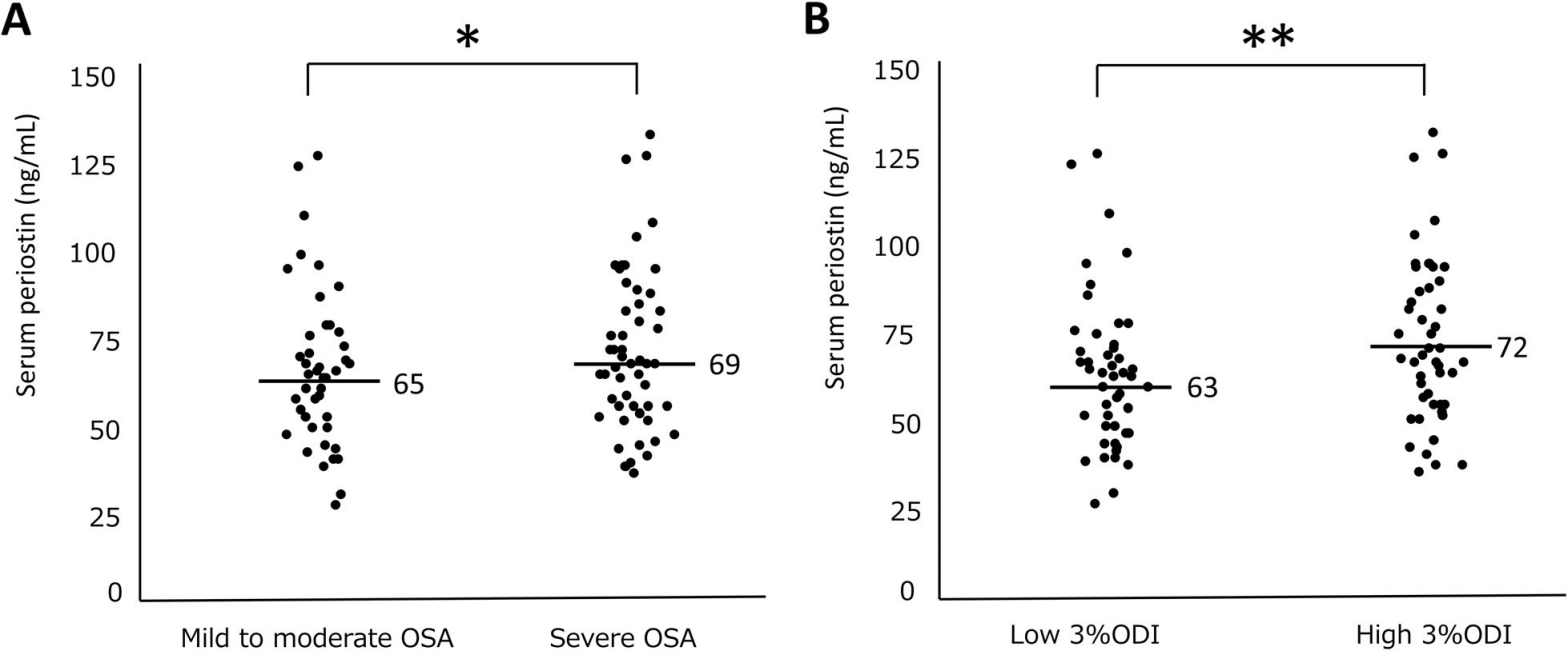
Associations between sleep metrics and serum periostin at baseline. **a** Severity of OSA and serum periostin (*p = 0.28 without adjustment for BMI, *p* = 0.07 with adjustment for BMI and **b** 3% ODI and serum periostin (**p = 0.03 without adjustment for BMI). High ODI was defined as 3% ODI ≥30.2/h, which was equivalent to the top 50% of the participants. OSA, obstructive sleep apnea; BMI, body mass index; 3% ODI, oxygen desaturation (≥3%) index

The associations between the serum periostin level and the clinical indices are presented in Table 2. Serum periostin level was positively correlated with age (*p* < 0.05) but was negatively correlated with BMI, albeit marginally (*p* = 0.055, Fig. 2). The serum periostin level did not differ between patients with severe OSA (*n* = 53) and those with mild to moderate OSA (*n* = 43) (*p* = 0.28), but it tended to be higher in patients with severe OSA when adjusted by BMI (*p* = 0.07) (Fig. 3a). Furthermore, serum periostin level was significantly higher in patients with high ODI (3% ODI ≥ 30.2/h) than in those with low ODI without adjustment (*p* = 0.03, Fig. 3b). The CT_90_, lowest oxygen saturation, and AI were not associated with serum periostin level, even after adjustment for BMI (data not shown). In addition, serum periostin level was not associated with the presence of major comorbidities, including COPD (*p* = 0.13), asthma (*p* = 0.32), and allergic rhinitis (*p* = 0.93) (Figure S1 in Additional file 1).

**Table 2:** Univariate linear regression analysis of the associations between clinical indices and serum periostin level^a^

Index	Estimate for serum periostin^a^ (95% CI)	***p*** − value
Age, years	3.9 × 10^−3^ (1.3 × × 10^−3^–6.6 × 10^− 3^)	< 0.01
BMI, kg/m^2^	−6.9 × 10^− 3^ (−14 × 10^− 3^–0.2 × 10^− 3^)	0.055
AHI, event/h	0.2 × 10^− 3^ (− 1.3 × 10^− 3^–1.6 × 10^− 3^)	0.82
CT_90_, %	−0.2 × 10^− 3^ (− 1.9 × 10^− 3^–1.4 × 10^− 3^)	0.73
Lowest oxygen saturation, %	2 × 10^− 4^ (− 3.4 × 10^− 3^–3.8 × 10^3^)	0.91
3% ODI, event/h	6.5 × 10^− 5^ (− 1.4 × 10^− 3^–1.5 × 10^− 3^)	0.92
Arousal index, event/h	3.8 × 10^− 4^ (− 1.3 × 10^− 3^–0.002)	0.66
Total cholesterol, mg/dL	−0.6 × 10^−3^ (− 1.5 × 10^− 3^–0.3 × 10^− 3^)	0.19
High-density lipoprotein, mg/dL	0.6 × 10^− 3^ (− 1.4 × 10^− 3^–2.7 × 10^− 3^)	0.53
Low-density lipoprotein, mg/dL	−0.3 × 10^− 3^ (− 1.3 × 10^− 3^–0.6 × 10^− 3^)	0.49
Triglycerides, mg/dL	−0.2 × 10^− 3^ (− 0.4 × 10^− 3^–0.09 × 10^− 3^)	0.22
Free fatty acids, mg/dL	0.09 × 10^− 3^ (− 0.1 × 10^− 3^–0.3 × 10^− 3^)	0.37
Blood glucose, mg/dL	1.6 × 10^− 3^ (0.4 × 10^− 3^–2.8 × 10^− 3^)	< 0.01
Hemoglobin A1c, %	35.3 × 10^− 3^ (1.8 × 10^− 3^–68.8 × 10^− 3^)	0.04
Urinary albumin–creatinine ratio^b^, mg/g	1.61 × 10^− 5^ (− 26.3 × 10^− 5^–29.6 × 10^− 5^)	0.91

^a^Log-transformed. ^b^Defined as urine albumin/creatinine

*CI* confidence interval, *BMI* body mass index, *AHI* apnea–hypopnea index, *CT*_*90*_ cumulative percentage of sleep time with percutaneous oxygen saturation < 90%, *ODI* oxygen desaturation index

### Cluster analysis and comparative analysis in patients with severe OSA

Subsequently, to comprehensively understand the associations among serum periostin levels, severe OSA, and comorbidities, cluster analysis was performed. This identified four clusters (Table 3 and Figure S2 and Supplementary Results in Additional file 1), including two clusters characterized by severe OSA (clusters 3 and 4). Compared with cluster 3, cluster 4 showed a high serum periostin level (87.1 ± 32.3 vs. 66.5 ± 21.8 ng/mL, *p* = 0.05) and a higher prevalence of the comorbidities: DM (*p* < 0.01), albuminuria (*p* < 0.01), hypertension (*p* = 0.03), and asthma (*p* = 0.01).

**Table 3:** Characteristics of each cluster

Index	Cluster (n)				***p***-value	
	1 (6)	2 (30)	3 (52)	4 (8)	All cluster	3 vs. 4
Age, years	47 ± 9	63 ± 9	59 ± 12	63 ± 8	0.01	0.33
Sex, male/female, n	6/0	23/7	40/12	5/3	0.43	0.38
Smoking, current/others, n	3/3	9/21	7/45	2/6	0.11	0.39
BMI, kg/m^2^	29.6 ± 2	27.1 ± 3.1	27.4 ± 4.6	30 ± 5.2	0.26	0.24
AHI, events/h	33 ± 33	29 ± 17	39 ± 20	51 ± 18	< 0.01	0.08
Severity of OSA^a^, mild–moderate/severe, n	5/1	22/8	15/37	1/7	< 0.01	0.33
CT_90_, %	12.7 ± 25.3	13.3 ± 19.1	12.3 ± 16.4	18.5 ± 21.1	0.85	0.22
Lowest oxygen saturation, %	76.5 ± 12.5	80.5 ± 7.3	79.2 ± 8.0	73.3 ± 9.5	0.14	0.1
3% ODI, events/h	31.2 ± 33.8	28.2 ± 17.3	35.6 ± 19.6	50.6 ± 19.7	0.04	0.06
High ODI^b^, +/−, n	1/5	9/21	31/21	7/1	< 0.01	0.13
Arousal index, events/h	21.5 ± 10.9	29.4 ± 13.9	40.6 ± 19.4	37.8 ± 19.1	0.03	0.66
Serum periostin, ng/mL	48.2 ± 14.7	67.1 ± 19.7	66.5 ± 21.8	87.1 ± 32.3	0.04	0.05
Serum periostin, high/low^c^, n	0/6	7/23	8/44	4/4	0.07	0.02
Blood eosinophils, /μL	204 (99–334)	202 (159–355)	225 (124–317)	245 (165–288)	0.86	0.68
Total cholesterol, mg/dL	228 ± 39	186 ± 32	195 ± 31	215 ± 41	0.03	0.11
High-density lipoprotein, mg/dL	44 ± 10	52 ± 13	55 ± 16	53 ± 14	0.27	0.69
Low-density lipoprotein, mg/dL	155 ± 33	107 ± 34	117 ± 25	126 ± 20	< 0.01	0.36
Triglycerides, mg/dL	190 ± 74	177 ± 75	152 ± 110	263 ± 266	0.05	0.04
Free fatty acids, mg/dL	409 ± 103	535 ± 177	460 ± 147	577 ± 151	0.03	0.04
Blood glucose, mg/dL	96 ± 14	112 ± 26	92 ± 11	130 ± 45	< 0.01	< 0.01
Hemoglobin A1c, %	5.8 ± 0.4	6.8 ± 1.0	5.8 ± 0.4	7.2 ± 1.1	< 0.01	< 0.01
Dyslipidemia, +/−, n	5/1	26/4	26/26	5/3	< 0.01	0.16
Diabetes mellitus, +/−, n	0/6	21/9	0/52	5/3	< 0.01	< 0.01
Hypertension, +/−, n	2/4	25/5	24/28	7/1	< 0.01	0.03
Urinary albumin–creatinine ratio^d^, mg/g	30.5 ± 5.5	6.4 ± 3.7	4.7 ± 2.7	228.3 ± 322.3	< 0.01	< 0.01
Albuminuria^e^, macro/micro/−, n	0/6/0	0/0/30	0/0/52	2/6/0	< 0.01	< 0.01
History of coronary artery disease, +/−, n	0/6	2/28	4/48	0/8	0.77	0.42
History of stroke, +/−, n	1/5	0/30	3/49	1/7	0.25	0.48
Chronic obstructive pulmonary disease, +/−, n	0/6	1/29	1/51	0/8	0.91	0.69
Asthma, +/−, n	1/5	0/30	1/51	2/6	< 0.01	0.01
Allergic rhinitis, +/−, n	0/6	4/26	11/41	1/7	0.51	0.57

Baseline data are presented as mean ± SD or numbers, except for eosinophils, which are presented as median (range)

^a^Severity of OSA was defined on the basis of the AHI, as follows: mild, ≥5 to < 15; moderate, ≥15 to < 30; or severe, AHI ≥30. ^b^Defined as 3% ODI ≥30.2/h, which was equivalent to the top 50% of all participants. ^c^Defined as serum periostin ≥87 ng/mL, the highest quintile. ^d^Defined as urine albumin/creatinine. ^e^A urinary albumin–creatinine ratio of ≥300 mg/g in men and women was considered as macroalbuminuria, and a value of ≥20 mg/g in men or ≥ 30 mg/g in women was considered as microalbuminuria

*BMI* body mass index, *AHI* apnea–hypopnea index, *CT*_*90*_ cumulative percentage of sleep time with percutaneous oxygen saturation < 90%, *ODI* oxygen desaturation index

The patients with severe OSA were divided into two groups, using a cutoff serum periostin level of 87 ng/mL, which was equivalent to the highest quintile of serum periostin among all participants. Compared with the severe OSA alone group (*n* = 41), the severe OSA with high periostin group (*n* = 12) exhibited significantly higher free fatty acid levels (*p* = 0.03) and prevalence of albuminuria (*p* = 0.04) (Table 4) and a trend for more frequent DM (*p* = 0.07) and asthma (*p* = 0.06).

**Table 4:** Characteristics of patients with severe OSA and high serum periostin

Index	Mild/moderate OSA^a^n = 43	Severe OSA^a^ alone n = 41 n = 41	Severe OSA^a^ + high periostin^b^ n = 12	***p***-value^**‡**^
Age, years	60 ± 10	58 ± 12	63 ± 9	0.16
Sex, male/female, n	33/10	31/10	10/2	0.57
Smoking, current/others, n	12/31	8/33	1/11	0.36
BMI, kg/m^2^	26.2 ± 3.5	28.9 ± 4.6	28.6 ± 3	0.83
AHI, events/h	18.8 ± 6.9	51.1 ± 17.5	50 ± 13.5	0.90
CT_90_, %	5.5 ± 8.0	21 ± 22.7	13.8 ± 14.8	0.59
3% ODI, events/h	17.5 ± 7.3	48.5 ± 18.6	46.1 ± 13.4	0.83
Lowest oxygen saturation, %	82.6 ± 7.6	76 ± 7.5	75.6 ± 8.4	0.98
Arousal index, events/h	21.8 ± 8.8	45.4 ± 17.8	41.8 ± 12.6	0.51
Serum periostin, ng/mL	64.7 ± 22.4	59.7 ± 13.9	102.7 ± 15.4	–
Blood eosinophils, /μL	202 (124–350)	219 (131–299)	240 (155–284)	0.92
Total cholesterol, mg/dL	195 ± 32	199 ± 37	189 ± 32	0.40
High-density lipoprotein, mg/dL	55 ± 16	53 ± 13	48 ± 15	0.30
Low-density lipoprotein, mg/dL	114 ± 34	121 ± 28	111 ± 21	0.22
Triglycerides, mg/dL	158 ± 90	175 ± 148	207 ± 117	0.49
Free fatty acids, mg/dL	480 ± 172	474 ± 147	581 ± 139	0.03
Blood glucose, mg/dL	100 ± 23	100 ± 23	113 ± 33	0.12
Hemoglobin A1c, %	6.2 ± 0.9	6.1 ± 0.8	6.5 ± 1.1	0.19
Dyslipidemia, +/−, n	15/28	16/25	7/5	0.24
Diabetes mellitus, +/−, n	14/29	7/34	5/7	0.07
Hypertension, +/−, n	22/21	29/12	8/4	0.79
Urinary albumin–creatinine ratio^c^, mg/g	10.5 ± 15.9	22.5 ± 89.4	89.4 ± 258.5	0.31
Albuminuria^d^, macro/micro/−, n	0/6/37	1/3/37	1/3/8	0.04^e^
Chronic obstructive pulmonary disease, +/−, n	1/41	0/41	1/11	0.06
Asthma, +/−, n	1/42	1/40	2/10	0.06
Allergic rhinitis, +/−, n	8/35	6/35	2/10	0.89

Baseline data are presented as mean ± SD or numbers, except for eosinophils, which were presented as median (range)

^a^Severity of OSA was defined on the basis of the AHI, as follows: mild, ≥5 to < 15; moderate, ≥15 to < 30; or severe, AHI ≥30

^b^Determined as high when the serum periostin level was ≥87 ng/mL (highest quintile)

^‡^*p-*values for severe OSA + high serum periostin vs. severe OSA alone

^c^Defined as urine albumin/creatininec

^d^Defined as positive if the urinary albumin–creatinine ratio was ≥20 mg/g in men or ≥ 30 mg/g in women

^e^Compared frequency of albuminuria

*OSA* obstructive sleep apnea, *BMI* body mass index, *AHI* apnea–hypopnea index, *CT*_*90*_ cumulative percentage of sleep time with percutaneous oxygen saturation < 90%, *ODI* oxygen desaturation index

**Fig. 4:**
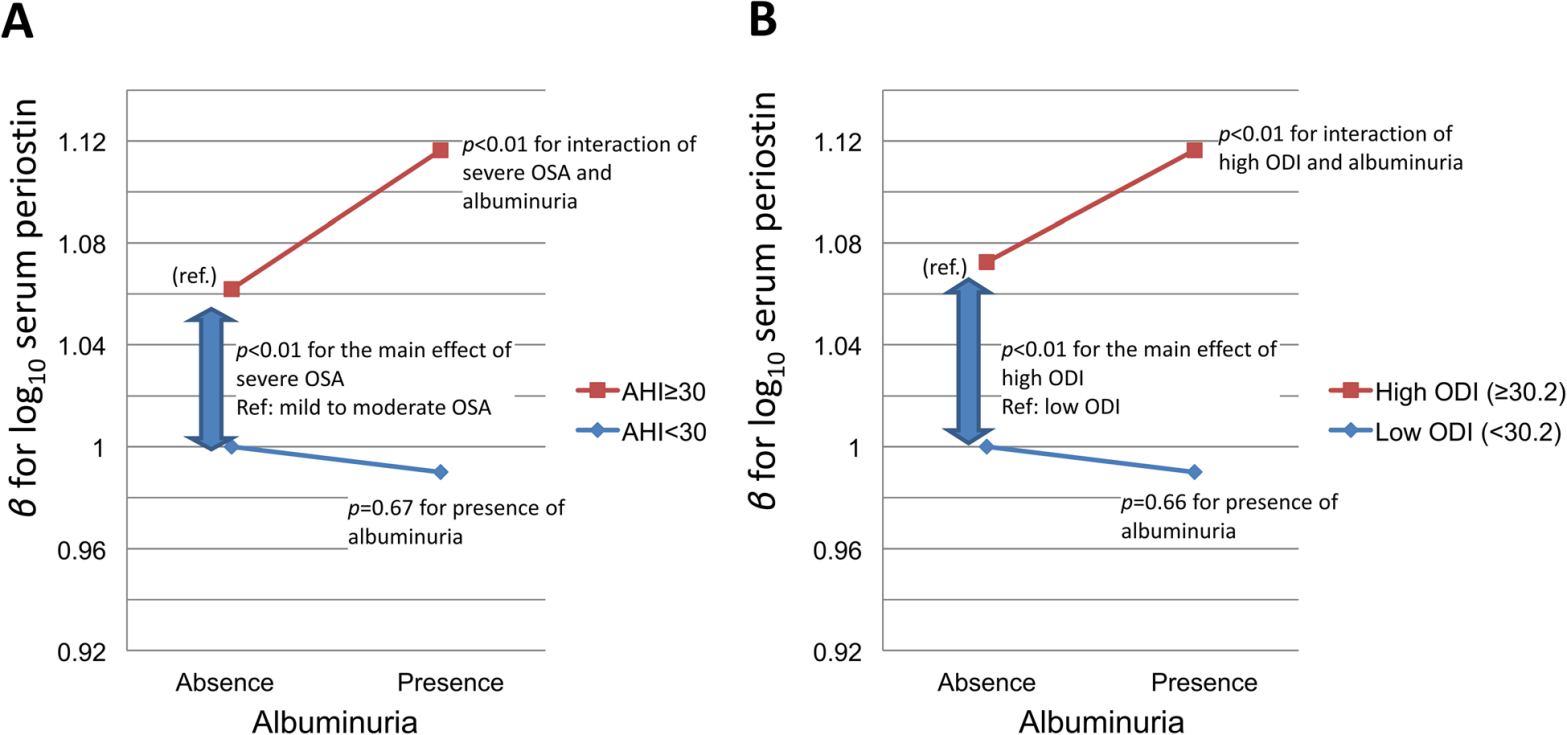
Positive interaction for serum periostin levels between severe OSA or high 3% ODI and albuminuria. **a** Severe OSA and presence of albuminuria and **b** high 3% ODI and presence of albuminuria. Albuminuria was considered present if the urinary albumin–creatinine ratio was ≥20 mg/g in men or ≥ 30 mg/g in women. High 3% ODI was defined as ≥30.2/h, which was equivalent to the top 50% of the participants. OSA, obstructive sleep apnea; AHI, apnea–hypopnea index; 3% ODI, oxygen desaturation (≥3%) index

Finally, among the entire study population, patients with severe OSA or high 3% ODI exhibited positive interactions with albuminuria for serum periostin levels (*p* < 0.01, Fig. 4a and b). No such interaction for high serum periostin level was observed in the associations between severe OSA and serum-free fatty acid or blood glucose (data not shown).

### Responsiveness to CPAP treatment in patients with severe OSA

Treatment with CPAP was completed successfully in 8 patients from the severe OSA with high periostin group and in 35 patients from the severe OSA alone group. In both groups, marked improvements in sleep metrics were observed (Table 5) and UACR decreased significantly after CPAP treatment, although this decrease was more marked in the severe OSA with high periostin group than that in the severe OSA alone group (− 71.4 ± 186.2 mg/g vs. − 12.9 ± 63.1 mg/g, *p* = 0.04). Significant decrease in the serum periostin level after CPAP treatment was seen in the severe OSA with high periostin group (*p* = 0.04) but not in the severe OSA alone group (*p* = 0.38). The serum periostin level did not decrease in subjects with moderate OSA (*p* = 0.75, Table S1 in Additional file 1).

**Table 5:** Clinical effects of CPAP in patients with severe OSA^a^ and high serum periostin^b^

Index	Severe OSA^a^ alone (***n*** = 35)			Severe + high periostin^b^ (***n*** = 8)		
	Baseline	3 M after CPAP	***p***-value	Baseline	3 M after CPAP	***p***-value
BMI, kg/m^2^	28.7 ± 4.8	28.9 ± 4.5	0.47	28.2 ± 3.5	28.6 ± 3.4	0.18
AHI, events/h	52.3 ± 18	3.4 ± 3.8	< 0.01	51.3 ± 15.9	5.1 ± 4.5	< 0.01
CT_90_, %	22.4 ± 23.7	0.2 ± 0.8	< 0.01	14 ± 6	0.7 ± 1.3	0.05
3% ODI, events/h	50.1 ± 19.5	2.8 ± 3.6	< 0.01	46.5 ± 16	4.5 ± 4.3	< 0.01
Arousal index, events/h	50 ± 17.7	18.9 ± 6.8	< 0.01	43.7 ± 14	20.2 ± 8.5	< 0.01
Lowest oxygen saturation, %	75.5 ± 7.8	91.1 ± 4.1	< 0.01	72.9 ± 8.5	89.5 ± 3.3	< 0.01
Serum periostin, ng/mL	60.1 ± 14	61.8 ± 15.5	0.38	104.1 ± 15.8	94 ± 10.8	0.04
Total cholesterol, mg/dL	199 ± 39	193 ± 38	0.21	201 ± 27	203 ± 29	0.58
High-density lipoprotein, mg/dL	52 ± 14	52 ± 12	0.92	53 ± 14	56 ± 15	0.05
Low-density lipoprotein, mg/dL	122 ± 29	119 ± 31	0.48	118 ± 19	123 ± 19	0.27
Triglycerides, mg/dL	179 ± 159	153 ± 95	0.12	204 ± 122	207 ± 95	0.79
Free fatty acids, mg/dL	464 ± 145	457 ± 171	0.81	609 ± 106	515 ± 116	0.25
Blood glucose, mg/dL	99 ± 23	96 ± 18	0.64	120 ± 37	101 ± 17	0.66
Hemoglobin A1c, %	6.1 ± 0.8	6.0 ± 0.7	0.62	6.8 ± 1.2	6.2 ± 0.6	0.26
Albuminuria^c^, macro/micro/−, n	1/3/31	0/8/27	0.16^e^	1/3/4	1/2/5	0.32^e^
Urinary albumin–creatinine ratio^d^, mg/g	25.6 ± 96.6	12.7 ± 34.2	< 0.01	132.1 ± 306.5	60.6 ± 121	< 0.01
Average CPAP use, min/day	304 ± 78		–	385 ± 70		0.01^f^
Percent days of CPAP use > 4 h, %	65 ± 29		–	75 ± 24		0.36^f^

Data are presented as mean ± SD or numbers

^a^Severe OSA was defined as OSA with AHI ≥30

^b^Determined as high when the serum periostin level was ≥87 ng/mL (highest quintile) at baseline

^c^Defined as positive if the urinary albumin–creatinine ratio was ≥20 mg/g in men or ≥ 30 mg/g in women

^d^Defined as urine albumin/creatinine

^e^Evaluated by the McNemar test, which examined the changes in the frequency of albuminuria following CPAP treatment

^f^Comparison of the severe OSA with high periostin group with the severe OSA alone group

*CPAP* continuous positive airway pressure, *OSA* obstructive sleep apnea, *BMI* body mass index, *AHI* apnea–hypopnea index; CT_90_, cumulative percentage of sleep time with percutaneous oxygen saturation < 90%, *ODI* oxygen desaturation index

## Discussion

To the best of our knowledge, this is the first study to demonstrate that serum periostin plays a role in severe OSA. Severe OSA, which is defined as AHI > 30, alone was not associated with serum periostin levels; this result may have been affected by the negative influence of BMI on serum periostin level. However, a high 3% ODI was significantly associated with serum periostin level. Notably, in the cluster and comparative analyses, an elevated serum periostin level was associated with albuminuria among patients who had severe OSA or high 3% ODI. These findings indicated that overweight/obese patients with elevated serum periostin level may comprise a specific subtype of severe OSA with albuminuria (Figure S3 in Additional file 1) and require careful follow-up.

Recently, heterogeneity has been recognized in OSA, particularly in severe OSA [29]. Previous studies that used cluster analysis demonstrated several severe OSA phenotypes, which exhibited similar AHI-based severity but different symptoms or frequencies of comorbidities [7, 8]. The present study identified four clusters that differed in age, sleep metrics, and laboratory data. Of the two distinct clusters of severe OSA, the cluster with comorbidities showed a trend of higher 3% ODI, compared with that in the cluster without comorbidities. This result was consistent with the previous findings that IH, which is represented by ODI, played a significant role in systemic inflammation [30] and was, in itself, a likely risk factor for comorbidities [31].

Periostin is secreted as a 93.3-kDa extracellular matrix protein [11]. Consistent with previous studies on patients with asthma and the general population [12–16], the present study showed that the serum periostin level was negatively associated with BMI, albeit marginally. This precluded us from using serum periostin as a simple marker to predict severe OSA. Therefore, we examined a severe OSA phenotype that was associated with high serum periostin, based on our findings in the cluster analysis and on previous experimental studies, which showed that the major pathologic features of OSA were associated with the upregulation of periostin. In particular, IH stimulation, as represented by the ODI, was shown to induce oxidative stress, production of reactive oxidative species, and activation of nuclear factor-κB pathways, which may result in the upregulation of periostin [32–34]. In the present study, high 3% ODI was significantly associated with the serum periostin level (Fig. 3b). Importantly, patients with severe OSA and elevated serum periostin level showed a decrease in serum periostin after CPAP treatment.

In the present study, significant associations among albuminuria, severe OSA, and high serum periostin level were observed consistently in both the cluster and comparative analyses. In some studies, periostin was shown to be upregulated in the renal tissues and in the urine of patients with diabetic nephropathy [24], as well as in mouse models of kidney injury [35]. However, no studies have shown elevation of serum periostin levels in patients with albuminuria. In addition, epidemiologic studies on the association of serum periostin levels with renal function have yielded inconsistent results [15, 36]. Meanwhile, albuminuria is one of the important comorbidities associated with OSA [27, 37], probably as a result of sleep-related pathophysiologic changes in the glomerulus [27]. In a mouse model of OSA, IH was shown to cause kidney injury accompanied by increased urinary albumin levels, possibly through glomerular hypertrophy, and increased expression of transforming growth factor-β1 and connective tissue growth factor [38]. On the basis of the fact that these are well-known upregulators of periostin expression [39], our present findings of high serum periostin levels in patients with severe OSA and albuminuria may reflect the synergistic effect of kidney injury and repetitive IH stimulation. Although serum periostin level was not associated with albuminuria alone, it had a positive interaction with albuminuria and severe OSA or high 3% ODI (Fig. 4). In conclusion, from a clinical standpoint, having high serum periostin levels despite being overweight/obese may be an identifier of a severe OSA with albuminuria subtype, which may benefit from CPAP treatment, as observed in this study.

The present study had several limitations. First, besides its retrospective nature, this study did not discuss certain severe comorbidities of OSA, such as arrhythmia and heart failure. This was due to an insufficient number of subjects with these comorbidities. Similarly, this study included few subjects with COPD, which is an important confounder of the severity of OSA and hypoxia [27]. However, even after excluding subjects with COPD, we confirmed that severe OSA was associated with high serum periostin level after adjustment for BMI and that high serum periostin level in severe OSA was a risk factor for albuminuria (*p* = 0.03, data not shown). Second, the presence of hypertension or dyslipidemia was defined according to the intake of antihypertensive or lipid-lowering medications. However, even if based on the diagnosis on self-reported disease (Table 1), we found that the prevalence of hypertension was similar in both the cluster and comparative analyses (data not shown). Third, the study included subjects who had already been treated with medications that may influence urinary albumin or lipid metabolism. This was inevitable, because many patients with OSA have these comorbidities that require medications [27]. Finally, four patients had comorbid asthma, and they exhibited numerically higher serum periostin level, compared with that in the remaining subjects (Figure S1 in Additional file 1). Even after excluding asthmatics from the analysis, the presence of albuminuria was seen in the severe OSA with high periostin group than in the severe OSA alone group (*p* = 0.03). Furthermore, the findings of the present study indicated that the presence of OSA in obese asthmatics should be suspected when the serum periostin level is elevated disproportionally to their BMI. Because OSA is a well-known comorbidity in obese patients with asthma [40] and contributes to poor asthma control [4, 41], serum periostin may assist in the management of this treatable trait in refractory asthma [42].

## Conclusions

In conclusion, patients with severe OSA were clustered into two groups, severe OSA with and without comorbidities. In addition, high serum periostin in patients with OSA, despite being overweight/obese, may be an indicator of severe OSA with comorbidities, particularly albuminuria.

## Supplementary information

**Additional file 1: Supplementary methods.** Definitions of the severity of obstructive sleep apnea (OSA) and of high/low for the other sleep parameters. The definitions severity of OSA and high/low sleep parameters in the current study. Cluster analysis. The detailed description of cluster analysis. Definitions of comorbidities. The detailed definition of the presence of major comorbidities in the current study. Supplementary results. Characteristics of four clusters obtained according to cluster analysis. Cluster 1 was characterized mainly by younger age, male population, dyslipidemia, and high BMI but low prevalence of severe OSA. Among the four clusters, the patients in this cluster had the lowest serum periostin levels. Cluster 2 composed mainly of subjects with mild to moderate OSA and those with abnormal glycometabolism. Clusters 3 and 4 were characterized by the accumulation of severe OSA cases. Albuminuria was present in eight subjects in cluster 4 and in no subject in cluster 3. Of the eight subjects, six were positive for microalbuminuria (four with DM) and two exhibited macroalbuminuria (one with DM). Compared with cluster 3, cluster 4 had higher mean ± SD serum periostin level (87.1 ng/mL ± 32.3 vs. 66.5 ± 21.8 ng/mL, *p* = 0.05); higher number of subjects with high serum periostin levels of ≥87 ng/mL, which was the highest quintile of serum periostin (*p* = 0.02); and higher prevalence of asthma. **Figure S1.** Serum periostin levels in patients with/without comorbidities. The presence of major comorbidities was not associated with serum periostin levels. The black bars and the numbers in the figure represent the mean serum periostin level. **Figure S2.** Distribution of the four clusters on the axes of apnea–hypopnea index and serum periostin levels. The details of each cluster are described in Table 3 and in the supplementary results (“Characteristics of four clusters obtained according to cluster analysis”). The encircled size represents the number of subjects in each cluster. The encircled number represents the cluster number in Table 3. **Figure S3.** Serum periostin level in OSA. Overweight/obesity has negative impacts on the serum periostin level in OSA, as observed in the general population. However, high intermittent hypoxia contributes to high serum periostin level. Furthermore, severe OSA and comorbid albuminuria show positive interactions for serum periostin level. A high serum periostin in patients with OSA despite being overweight/obese may indicate a phenotype of OSA (i.e., severe OSA with albuminuria). **Table S1.** Clinical effects of CPAP in patients with moderate OSA. The serum periostin level did not decrease in subjects with moderate OSA after CPAP treatment (*p* = 0.75).

## Data Availability

The datasets analyzed during the current study are not publicly available, because the consent obtained from the participants specified that the data can be used only for research purposes at our institution. The datasets can only be available from the corresponding author upon reasonable request and after the approval of the ethics committee of Kyoto University.

## Abbreviations

AHI: Apnea–hypopnea index
AI: Arousal index
BMI: Body mass index
COPD: Chronic obstructive pulmonary disease
CPAP: Continuous positive airway pressure
CT_90_: Cumulative percentage of sleep time with percutaneous oxygen saturation < 90%
DM: Diabetes mellitus
3% ODI: Oxygen desaturation (≥3%) index
OSA: Obstructive sleep apnea
PSG: Polysomnography
Th2: T helper type 2
UACR: Urine albumin–creatinine ratio
