# Supplementary material for: Role of serum periostin in severe obstructive sleep apnea with albuminuria: an observational study
Source: Respir Res. 2020 Jun 9;21:143. doi: 10.1186/s12931-020-01413-0 (PMC7285606; doi:10.1186/s12931-020-01413-0)
Supplement: Supplementary file 1 — Additional file 1: Supplementary methods. Definitions of the severity of obstructive sleep apnea (OSA) and of high/low for the other sleep parameters. The definitions severity of OSA and high/low sleep parameters in the current study. Cluster analysis. The detailed description of cluster analysis. Definitions of comorbidities. The detailed definition of the presence of major comorbidities in the current study. Supplementary results. Characteristics of four clusters obtained according to cluster analysis. Cluster 1 was characterized mainly by younger age, male population, dyslipidemia, and high BMI but low prevalence of severe OSA. Among the four clusters, the patients in this cluster had the lowest serum periostin levels. Cluster 2 composed mainly of subjects with mild to moderate OSA and those with abnormal glycometabolism. Clusters 3 and 4 were characterized by the accumulation of severe OSA cases. Albuminuria was present in eight subjects in cluster 4 and in no subject in cluster 3. Of the eight subjects, six were positive for microalbuminuria (four with DM) and two exhibited macroalbuminuria (one with DM). Compared with cluster 3, cluster 4 had higher mean ± SD serum periostin level (87.1 ng/mL ± 32.3 vs. 66.5 ± 21.8 ng/mL, p = 0.05); higher number of subjects with high serum periostin levels of ≥87 ng/mL, which was the highest quintile of serum periostin (p = 0.02); and higher prevalence of asthma. Figure S1. Serum periostin levels in patients with/without comorbidities. The presence of major comorbidities was not associated with serum periostin levels. The black bars and the numbers in the figure represent the mean serum periostin level. Figure S2. Distribution of the four clusters on the axes of apnea–hypopnea index and serum periostin levels. The details of each cluster are described in Table 3 and in the supplementary results (“Characteristics of four clusters obtained according to cluster analysis”). The encircled size represents the number of subjects in [file 12931_2020_1413_MOESM1_ESM.docx]

**Unmarked R2 Additional file 1**

**Additional files**

**Supplementary Methods**

**Definitions of the severity of obstructive sleep apnea (OSA) and of high/low for the other sleep parameters**

Patients with an apnea–hypopnea index (AHI) ≥5 were defined as having OSA, and were classified as having either mild (AHI, <15), moderate (AHI, 15 to less than 30), or severe (AHI ≥30). The cumulative percentage of sleep time with percutaneous oxygen saturation (SpO_2_) <90% (CT_90_) ≥5.6 was defined as high CT_90_, an oxygen desaturation (≥3%) index (3% oxygen desaturation index (ODI)) ≥30.2 was defined as high ODI, and an arousal index (AI) ≥31.9 was defined as high AI; these were the thresholds of the top 50% for each variable in this study.

**Cluster analysis**

For Ward’s hierarchical cluster analysis, the following variables that were measurable in daily practice and essential for the management of OSA and its comorbidities were first categorized: sex, age, body mass index (BMI), smoking status (current), presence of severe OSA (AHI ≥30), diabetic mellitus (DM), albuminuria, hypertension, and dyslipidemia. According to principal component analysis, these variables were reduced to four factors: presence of DM, severe OSA, albuminuria, and hypertension. Accordingly, cluster analysis was performed using these four variables.

**Definitions of comorbidities**

The presence of hypertension was defined as the usage of antihypertensive medications, and dyslipidemia as the use of lipid-lowering drugs unless specifically stated. Diabetes mellitus was considered present in patients with a fasting plasma glucose ≥126 mg/dL and hemoglobin A1c (HbA1c) ≥6.5%. HbA1c was defined by the Japan Diabetes Society and converted into that defined by the National Glycohemoglobin Standardized Program (1). Participants with a smoking history of at least 10 pack-years and airflow limitation were considered to have chronic obstructive respiratory disease. Airflow limitation was defined as forced expiratory volume in 1 second/forced vital capacity ratio <0.7. The presence of asthma and allergic rhinitis were determined based on the medical history.

**Supplementary Results**

**Characteristics of four clusters obtained according to cluster analysis**

Cluster 1 was characterized mainly by younger age, male population, dyslipidemia, and high BMI but low prevalence of severe OSA. Among the four clusters, the patients in this cluster had the lowest serum periostin levels. Cluster 2 composed mainly of subjects with mild to moderate OSA and those with abnormal glycometabolism. Clusters 3 and 4 were characterized by the accumulation of severe OSA cases. Albuminuria was present in eight subjects in cluster 4 and in no subject in cluster 3. Of the eight subjects, six were positive for microalbuminuria (four with DM) and two exhibited macroalbuminuria (one with DM). Compared with cluster 3, cluster 4 had higher mean ± SD serum periostin level (87.1 ng/mL ± 32.3 vs. 66.5 ± 21.8 ng/mL, *p* = 0.05); higher number of subjects with high serum periostin levels of ≥87 ng/mL, which was the highest quintile of serum periostin (*p* = 0.02); and higher prevalence of asthma.

**Figure S1** Serum periostin levels in patients with/without comorbidities


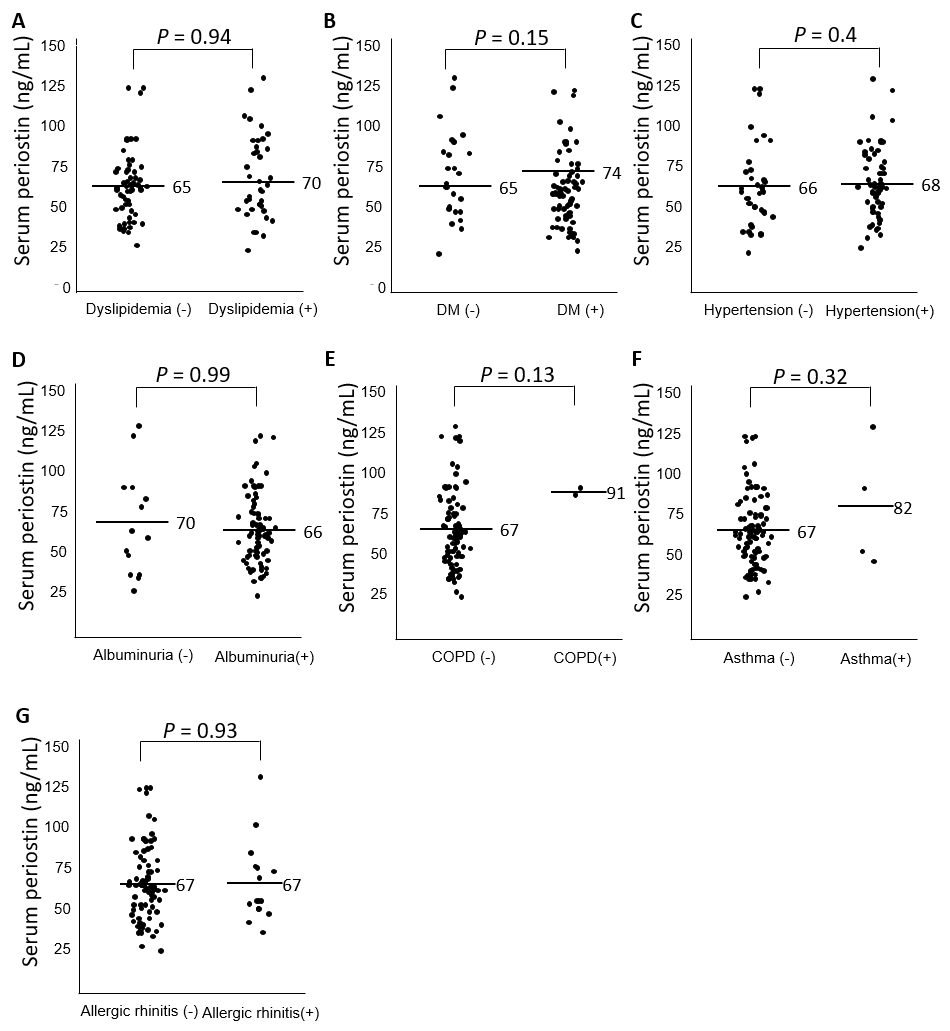


The black bars and the numbers in the figure represent the mean serum periostin level.

*P*-value was evaluated for log-transformed serum periostin, using t-test. Albuminuria was considered present if urinary albumin–creatinine ratio (UACR) was ≥20 mg/g in males or ≥30 mg/g in females. Definitions of other comorbidities are described in the Supplementary Methods.

DM, diabetic mellitus; COPD, chronic obstructive pulmonary disease.

**Figure S2** Distribution of the four clusters on the axes of apnea–hypopnea index and serum periostin levels


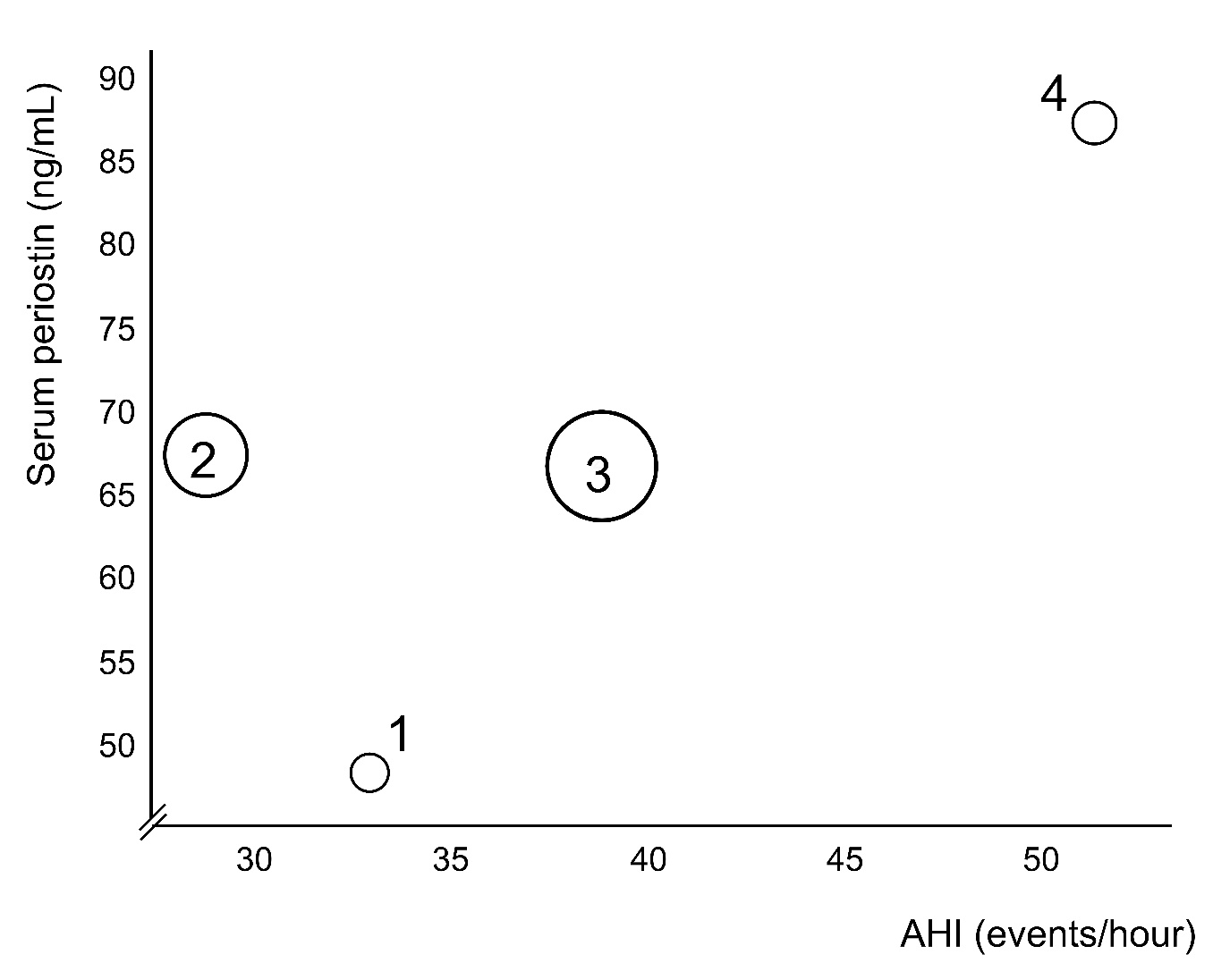


The encircled size represents the number of subjects in each cluster. The encircled number represents the cluster number in Table 3.

AHI, apnea–hypopnea index

**Figure S3** Serum periostin level in OSA


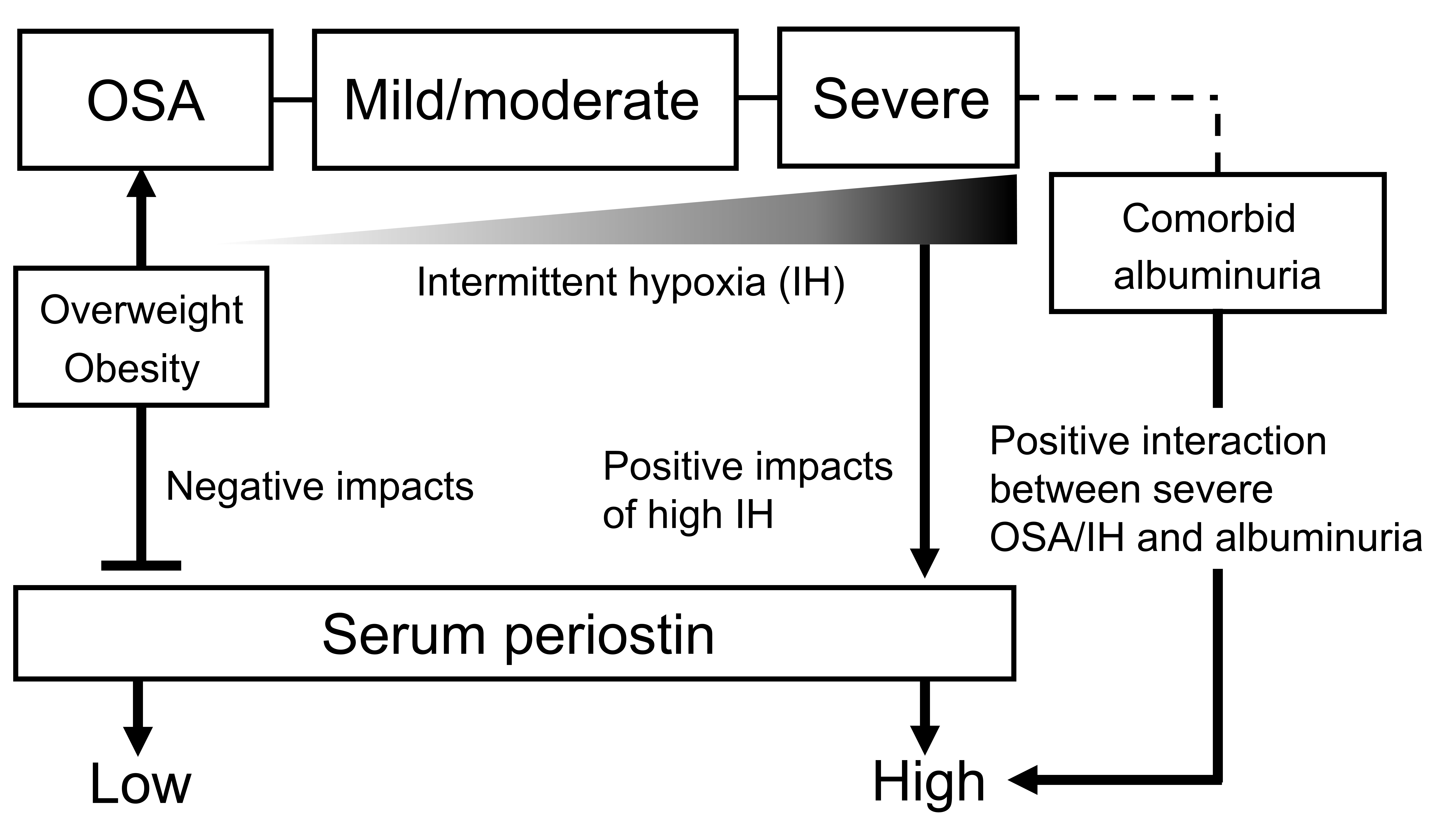


Overweight/obesity has negative impacts on serum periostin level in OSA, as observed in general population. However, high intermittent hypoxia contributes to high serum periostin level. Furthermore, severe OSA and comorbid albuminuria showed positive interactions for serum periostin level. A high serum periostin in patients with OSA despite being overweight/obese may indicate a phenotype of OSA, i.e., severe OSA with albuminuria.

Severity of OSA was defined as follows: mild to moderate, 30 > AHI ≥ 5 and severe, AHI ≥ 30. Albuminuria was defined as positive if urinary albumin–creatinine ratio was ≥20 mg/g in males or ≥30 mg/g in females.

OSA, obstructive sleep apnea; IH, intermittent hypoxia.

**Table S1** Clinical effects of CPAP in patients with moderate OSA*

|  | **Participants with moderate OSA***  **and completed CPAP therapy (n = 20)** | | |
| --- | --- | --- | --- |
|  | **Baseline** | **3M after CPAP** | ***P*-value** |
| BMI, kg/m^2^ | 27.3 ± 2.9 | 27.2 ± 2.8 | 0.83 |
| AHI, events/h | 22.6 ± 2.3 | 4.3 ± 3.1 | <0.01 |
| CT_90_, % | 8.1 ± 9.7 | 1.5 ± 6 | <0.01 |
| 3% ODI, events/h | 21.6 ± 4.3 | 3.8 ± 3.5 | <0.01 |
| Lowest oxygen saturation, % | 80.1 ± 8.4 | 90.3 ± 4.1 | <0.01 |
| Arousal Index, events/h | 21.8 ± 9.9 | 17.4 ± 7.7 | <0.01 |
| Serum periostin, ng/mL | 57.2 ± 16.3 | 58.1 ± 18 | 0.75 |
| Total cholesterol, mg/dL | 201 ± 33 | 194 ± 33 | 0.04 |
| High-density lipoprotein, mg/dL | 53 ± 16 | 52 ± 14 | 0.66 |
| Low-density lipoprotein, mg/dL | 119 ± 33 | 117 ± 28 | 0.62 |
| Triglycerides, mg/dL | 180 ± 100 | 162 ± 64 | 0.19 |
| Free fatty acids, mg/dL | 484 ± 182 | 565 ± 151 | 0.12 |
| Blood glucose, mg/dL | 99 ± 19 | 102 ± 25 | 0.46 |
| Hemoglobin A1c, % | 6.3 ± 0.8 | 6.4 ± 1.1 | 0.97 |
| Albuminuria^†^, macro/micro/-, n | 0/4/16 | 0/8/11 | 0.03^§^ |
| Urinary albumin–creatinine ratio^‡^, mg/g | 14.2 ± 21.3 | 10.1 ± 12.5 | 0.03 |
| Average CPAP use, min/day | 281 ± 66 | | - |
| Days of CPAP use >4 h, % | 51 ± 29 | | - |

Data are presented as the mean ± SD or numbers.

*Among participants with moderate OSA, those with AHI ≥20 were treated with CPAP according to the health insurance system in Japan. No urinary data at follow-up was obtained for one patient in this group ^†^ Defined as positive if the urinary albumin–creatinine ratio was ≥20 mg/g in men or ≥30 mg/g in women ^‡^Defined as urine albumin/creatinine ^§^Evaluated by the McNemar test, which examined the changes in the frequency of albuminuria following CPAP treatment

CPAP, continuous positive airway pressure; OSA, obstructive sleep apnea; BMI, body mass index; AHI, apnea hypopnea index; CT_90_, cumulative percentage of sleep time with percutaneous oxygen saturation < 90%; ODI, oxygen desaturation index.

**Additional reference**

1. Seino Y, Nanjo K, Tajim N, Kadowaki T, Kashiwagi A, Araki E, et al. Report of the committee on the classification and diagnostic criteria of diabetes mellitus. *J Diabetes Investig* 2010;1:212–228.
